# Digoxin absorption decreased independently of P-gp activity in rats with irinotecan-induced gastrointestinal damage

**DOI:** 10.1186/s40780-021-00207-w

**Published:** 2021-07-01

**Authors:** Toshiaki Tsuchitani, Takeshi Akiyoshi, Ayuko Imaoka, Hisakazu Ohtani

**Affiliations:** Division of Clinical Pharmacokinetics, Keio University Faculty of Pharmacy, 1-5-30, Shibakoen Minato-ku, Tokyo, 105-8512 Japan

**Keywords:** Drug absorption, P-gp, Gastrointestinal damage, Digoxin, Clarithromycin

## Abstract

**Background:**

Irinotecan (CPT-11) is clinically known to cause severe diarrhea and gastrointestinal damage. Recently, we have reported that CPT-11-induced gastrointestinal damage is associated with the upregulation of intestinal P-glycoprotein (P-gp) expression and decreased absorption of its substrate, dabigatran etexilate (DABE), using a rat model. However, the P-gp activity or its contribution to the decreased absorption remains unclear. The aim of this study was to quantitatively evaluate how P-gp activity changes in rats with CPT-11-induced gastrointestinal damage, as assessed by the absorption of digoxin (DGX), a typical P-gp substrate.

**Methods:**

Male Sprague-Dawley rats were intravenously administered CPT-11 at a dose of 60 mg/kg/day for 4 days to induce gastrointestinal damage. Then, the rats were administered DGX orally (40 μg/kg), after some of them were orally administered clarithromycin (CAM; 10 mg/kg), a P-gp inhibitor. DGX (30 μg/kg) was administered intravenously to determine the bioavailability (BA). The rats’ DGX plasma concentration profiles were determined using LC-MS/MS.

**Results:**

CPT-11 treatment decreased the maximum concentration (C_max_) and area under the plasma concentration-time curve (AUC_po_) of DGX, which does not contradict to the DABE study. Although in the CPT-11-treated group the BA of DGX was significantly decreased to 40% of the control value, CAM did not affect the BA of DGX in the CPT-11-treated group.

**Conclusions:**

Increased P-gp expression in rats with CPT-11-induced gastrointestinal damage is not necessarily associated with increased P-gp activity or contribution to the drug absorption in vivo. The decreased DGX absorption observed in this study might be attributable to other factors, such as a reduction in the absorptive surface area of the gastrointestinal tract.

## Background

Irinotecan (CPT-11) is a topoisomerase-I inhibitor, which has been widely used as antineoplastic agent in the clinical setting since the late 1980s [1]. After being intravenously administered, CPT-11 is metabolized into its active metabolite, SN-38, by carboxylesterase, and then its glucuronide conjugate (SN-38G; SN-38 glucuronide) is inactivated by UDP-glucuronosyltransferase 1A1 [2, 3]. CPT-11, SN-38, and SN-38G are excreted into bile by various transporters, including P-glycoprotein (P-gp) [4–6]. SN-38G is deconjugated by the β-glucuronidases belonging to the intestinal microflora, resulting in SN-38 being released into the intestinal tract. As SN-38 induces gastrointestinal damage, which is sometimes life-threatening, intestinal toxicity is the primary dose-limiting toxicity of CPT-11 [7].

Drug absorption from the intestinal tract is affected by various factors, including gastrointestinal damage induced by anticancer drugs. In patients with chemotherapy-induced diarrhea, the absorption of lactulose, a marker of paracellular absorption, is increased, whereas the absorption of vitamin A, which requires intracellular processes to be absorbed, is decreased [8]. These findings indicate that the tissue damage induced in the intestinal tract by anticancer drugs essentially involves the atrophication of the intestinal villi and intracellular space expansion. Besides alterations in passive absorption, we have reported that in rats the intestinal expression levels of transporters, such as peptide transporter 1 (Pept1), P-gp, and breast cancer resistance protein (Bcrp), are also affected by anticancer drugs [9, 10]. Previously, we have demonstrated in rats that 5 days’ treatment with 5-FU (30 mg/kg/day) resulted in the 15-fold upregulation of P-gp expression and a 2.6-fold increase in Bcrp expression in the intestines, as demonstrated by Western blotting [9]. We have also shown in rats in which gastrointestinal damage was induced using intravenous CPT-11 (60 mg/kg/day for 4 consecutive days) that the intestinal expression of P-gp was significantly increased to 2- to 5- fold higher than the control level [10]. In addition, the bioavailability (BA) of dabigatran etexilate (DABE), a P-gp substrate, decreased 6.25-fold, suggesting that the upregulation of P-gp was responsible for the reduced BA of DABE. However, the changes in intestinal P-gp activity that occur in CPT-11-treated rats are yet to be elucidated.

The aim of this study was to quantitatively evaluate the absorption of digoxin (DGX), a typical probe for P-gp activity, in rats that had been treated with CPT-11. The rats were intravenously administered CPT-11 to induce gastrointestinal damage, and then they were orally administered DGX with or without oral clarithromycin (CAM), a P-gp inhibitor, to determine the pharmacokinetic (PK) parameters of DGX.

## Materials and methods

### Chemical reagents

CPT-11 (7-ethyl-10-[4(− 1-piperidino)-1-piper-idino] carbonyloxy camptothecin; Sawai Pharmaceutical Co., Osaka, Japan), DGX (Tokyo Chemical Industry Co., Tokyo, Japan), and CAM (Wako Pure Chemical Industries, Osaka, Japan) were commercially purchased and used in this study. Unless otherwise noted, all other reagents were commercially purchased from Nacalai Tesque Inc., Tokyo, Japan.

### Development of a rat model of irinotecan-induced gastrointestinal damage

Seven-week-old male Sprague-Dawley rats (Sankyo Labo Service Corporation, Tokyo, Japan), weighing 200–250 g, were housed under standard conditions for 1 week before the CPT-11 treatment. Throughout the experiments, the rats were housed individually and allowed free access to food and water.

Some of the rats were intravenously administered 60 mg/kg of CPT-11 via the tail vein under isoflurane anesthesia once daily for 4 days (the CPT-11 treated group). Three mL/kg saline was administered as a control (the control group). Body weight, food intake, the fecal count, and fecal weight were monitored during the 4-day administration period. On the day after the final dose of CPT-11 was administered (day 5), the state of the rats’ feces and the perianal staining were examined and scored according to the diarrhea score criteria (Table 1) [10–14].

**Table 1:** Diarrhea score criteria

Score	Condition of feces and perianal staining
0	normal: normal feces or absent
1	slight diarrhea: slightly wet and soft feces
2	moderate diarrhea: wet and unformed feces with moderate perianal staining of the coat
3	severe diarrhea: watery feces with severe perianal staining of the coat

### Pharmacokinetics of digoxin

On day 4, the jugular vein was cannulated under isoflurane anesthesia, and the rats were fasted overnight before the experiment. On day 5, 40 μg/kg of DGX solution (0.05 mg/mL Digosin® Elixir, Chugai Pharmaceutical Co.) was administered orally. Ten mg/kg CAM, suspended in 0.5% carboxymethyl cellulose (CMC), was orally administered 5 min before the DGX in the DGX + CAM group. One hundred and fifty-μL blood samples were collected from the jugular vein at 7.5, 15, 30, 60, 120, 180, 360, 720, and 1440 min after the oral administration of DGX. To determine the BA of DGX, 30 μg/kg of DGX (0.25-mg Digosin® injections; Chugai Pharmaceutical Co., Tokyo, Japan) was intravenously injected via the canula on day 6. In the DGX alone and DGX + CAM groups, CMC solution and a CAM suspension, respectively, were orally administered 5 min before the DGX injection. One hundred and fifty-μL blood samples were again collected from the jugular vein at 2, 5, 10, 20, 30, 60, 120, 180, 360, and 720 min after the intravenous administration of DGX. Each blood sample was kept on ice and centrifuged at 3000×*g* for 10 min at 4 °C. The plasma was separated and stored at − 20 °C until it was analyzed.

### LC-MS/MS analysis

Ten μL of internal standard solution (25 ng/mL digitoxin) was spiked into a glass microtube and then evaporated to dryness, before 50 μL of plasma sample was added. Eight different standard solutions of DGX, with concentrations ranging from 0.03 to 100 ng/mL, were prepared from blank plasma. To each sample, 500 μL of methyl-*t*-butyl ether was added, before the sample was vortexed and cooled under 4 °C for 15 min. The samples were centrifuged at 3000×*g* for 10 min at 4 °C, and 300 μL of the supernatant was evaporated at 50 °C. The residue was dissolved in 100 μL of the mobile phase, and 10 μL of the sample was subjected to LC-MS/MS analysis, as described below.

The DGX concentration was determined using an LC-MS/MS system, consisting of a controller (CBM-20A, Shimadzu, Kyoto, Japan), a pump (LC-20 AD, Shimadzu), a triple quadrupole mass spectrometer (LCMS-8050, Shimadzu), an octadecylsilane column (particle diameter: 5 μm, internal diameter: 2.0 mm, length: 150 mm; Cosmosil, 5C_18_-MS-II; Nacalai Tesque, Kyoto, Japan), and a column oven (CTO-20 AC, Shimadzu) set at 40 °C. The mobile phase was prepared as an equivalent mixture of aqueous and acetonitrile with 0.1% formic acid, and the flow rate was set at 0.3 mL/min. The MS/MS analysis was conducted in negative-ion mode using electrospray ionization. The DGX and digitoxin ion pairs used for the multiple reaction monitoring in this study were 825.45/779.30 and 809.45/763.25, respectively. The concentrations of the plasma samples were determined using a standard curve obtained via the internal standard method based on the peak area ratio.

### Pharmacokinetic analysis

AUC (area under the plasma concentration curve) and AUMC (area under the first moment curve) values were calculated using the trapezoidal method from t = 0 to the final blood sampling point. To assess the plasma concentration of DGX at the time of the intravenous administration of the drug, the y-intercept was determined by extrapolating the line passing through the concentrations at t = 0.033 and 0.083 (hr). The elimination rate constant (k_e_) was defined via log-linear regression during the elimination phase after oral administration. PK parameters were calculated using the following equations:

$$ \mathrm{MRT}=\mathrm{AUMC}/\mathrm{AUC} $$

$$ \mathrm{MAT}={\mathrm{MRT}}_{\mathrm{po}}-{\mathrm{MRT}}_{\mathrm{iv}} $$

$$ \mathrm{BA}=\left({\mathrm{AUC}}_{\mathrm{po}}/{\mathrm{D}}_{\mathrm{po}}\right)/\left({\mathrm{AUC}}_{\mathrm{iv}}/{\mathrm{D}}_{\mathrm{iv}}\right)\times 100 $$

$$ {\mathrm{Vd}}_{\mathrm{iv}}={\mathrm{D}}_{\mathrm{iv}}/{\mathrm{C}}_{0,\mathrm{iv}} $$

### Statistical analysis

The significance of differences in body weight, food intake, fecal count, fecal mass, or the diarrhea score between the control group and the CPT-11-treated group were determined using the Student’s *t*-test. The significance of differences in PK parameters was determined by two-way ANOVA followed by Holm’s multiple comparisons test. *P*-values of < 0.05 were considered statistically significant.

## Results

### Irinotecan-induced gastrointestinal damage in rats

**Fig. 1:**
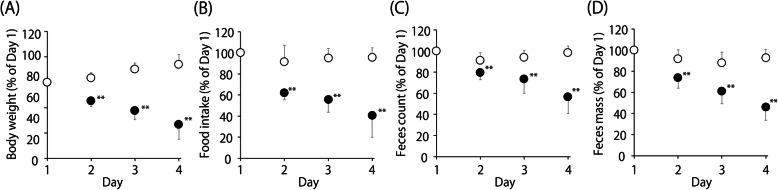
The effects of 4 days’ CPT-11 treatment in rats. The panels show the rates of change in body weight (**a**), food intake (**b**), fecal count (**c**), and fecal mass (**b**) throughout the CPT-11 treatment. The open circles represent the control group (n = 14, the intravenous administration of 3 mL/kg saline). The closed circles represent the CPT-11-treated group (*n* = 14, the intravenous administration of 60 mg/kg CPT-11). Data are shown as the mean ± SD. **p < 0.01

After the intravenous administration of CPT-11 for 4 days, significant reductions in body weight, food intake, the number of feces, and fecal mass 4 were observed (Fig. 1). The mean diarrhea score of the CPT-11-treated group on day 4 was 2.2 while that of the control group was zero (Table 2). These results were comparable with those described in previous reports [10–14].

**Table 2:** Diarrhea score after the administration of CPT-11 for 4 days

Group	Score				Mean
	0	1	2	3	
Control (n = 14)	14	0	0	0	0
CPT-11-treated (n = 14)	1	3	2	8	2.2^**^

**p < 0.01

### Pharmacokinetic analysis of digoxin

**Fig. 2:**
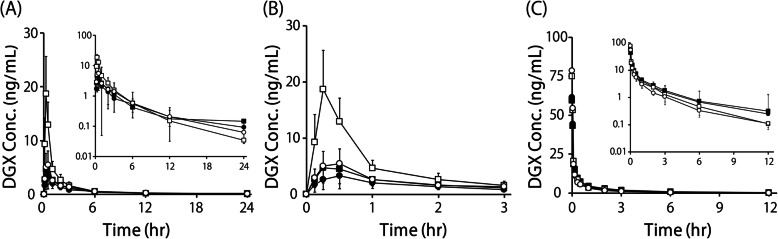
Plasma concentration-time curves of DGX after its oral administration at 40 μg/kg (**a**), (**b**) or its intravenous administration at 30 μg/kg (**c**). The insets in (**a**) and (**c**) show semi-logarithmic plots. (**b**) shows the enlarged figure of (**a**) until 3 h after oral administration of DGX. The open and closed circles represent the control and CPT-11-treated groups, respectively. The circles and squares represent the DGX alone and DGX + CAM treated groups, respectively. Data are shown as the mean ± SD. (*n* = 7)

In the CPT-11 treated group, the C_max_, AUC_po_, and BA values of DGX were markedly decreased to 47, 22, and 38% of the control values, respectively, when DGX was administered alone (Fig. 2a and b, Table 3). The MRT_po_ and MAT of the CPT-11 treated group were prolonged by 34 and 35%, respectively, compared with those of the control group (Table 3). CPT-11 treatment did not affect the k_e, po_ or k_e, iv_ value of DGX (Tables 3 and 4).

**Table 3:** PK parameters of DGX after it was orally administered alone or coadministered with CAM after CPT-11 treatment

	C_max_	AUC_po_	MRT_po_	MAT	BA	ke_po_
	(ng/mL)	(hr・ng/mL)	(hr)	(hr)	(%)	(1/h)
Control						
DGX alone	6.43 ± 2.75	14.4 ± 3.34	4.09 ± 0.75	2.55 ± 0.80	66.1 ± 11.1	0.187 ± 0.053
DGX + CAM	19.1 ± 6.39^*^	22.8 ± 7.73	2.55 ± 0.32^*^	0.885 ± 0.35^*^	92.3 ± 11.4^*^	0.224 ± 0.067
CPT-11						
DGX alone	3.40 ± 2.24	11.3 ± 2.84	5.50 ± 1.44	3.45 ± 1.27	40.8 ± 16.7^*^	0.166 ± 0.030
DGX + CAM	5.03 ± 2.86^#^	13.1 ± 3.27	4.95 ± 1.68^#^	2.79 ± 1.42^#^	42.9 ± 15.7^#^	0.155 ± 0.054

Data are shown as the mean ± SD. (n = 7); *p < 0.05 vs. DGX alone in the control group; #*p* < 0.05 vs. DGX + CAM in the control group

**Table 4:** PK parameters of DGX after it was intravenously administered alone or coadministered with oral CAM after CPT-11 treatment

	AUC_iv_	Vd_iv_	k_e, iv_	CL_iv_
	(hr・ng/mL)	(L/g)	(1/h)	(mL/hr)
Control				
DGX alone	16.9 ± 3.88	410 ± 98.4	0.181 ± 0.070	533 ± 109
DGX + CAM	19.5 ± 5.34	432 ± 113	0.235 ± 0.065	483 ± 110
CPT-11				
DGX alone	22.2 ± 4.59	517 ± 127	0.186 ± 0.043	361 ± 82.6^*^
DGX + CAM	23.9 ± 5.55	553 ± 177	0.162 ± 0.051	329 ± 71.4

Data are shown as the mean ± SD. (n = 7); *p < 0.05 vs. DGX alone in the control group

In the control group, CAM markedly increased the C_max_, AUC_po_, and BA values of DGX to 297, 158, and 140%, respectively. In the CPT-11-treated group, the C_max_, AUC_po_, and BA of DGX were slightly increased to 148, 116, and 105%, respectively, by CAM treatment. CAM did not affect the PK parameters of DGX after its intravenous administration in the control or CPT-11 treated group (Fig. 2c, Table 4).

Two-way ANOVA revealed statistically significant effects of CPT-11 on all of the PK parameters of DGX, except C_max_ and k_e, iv_. There were main effects of CAM on BA, AUC_po_, MRT_po_, and MAT. The interaction between CPT-11 treatment and CAM treatment had statistically significant effects on the BA, AUC_po_, MRT_po_, and AUC_iv_ of DGX.

## Discussion

After the administration of DGX alone, the C_max_, AUC_po_, and BA values of DGX were significantly lower in the CPT-11-treated group than in the control group, and these findings seem to be consistent with the results of our previous study of DABE [10]. However, CAM did not significantly affect the absorption kinetics of DGX in rats treated with CPT-11. This observation might indicate that P-gp did not play an important role in decreasing the absorption of DGX in the CPT-11-treated group. In the absence of CAM, CPT-11 treatment decreased the BA of DGX to 62%, which is consistent with the findings of our previous study, in which the BA of acetaminophen was significantly reduced by CPT-11 treatment indicating the decreased effective absorbing area [10]. Namely, since P-gp extrudes substrate molecules from inside epithelial cells, [15] the observed activity of P-gp also depends on the amount of substrate absorbed into these cells. The findings of the current study indicate that the upregulation of P-gp expression does not always lead to decreased absorption of P-gp substrates under certain conditions, such as gastrointestinal mucositis induced by antineoplastic agents. It is worth discussing whether this phenomenon is DGX-specific or not. Our preliminary experiment using DABE as an alternative probe of P-gp failed to find the difference in the effect of CAM on the pharmacokinetics of DABE between control and CPT-11-treated rats (unpublished observation), although the expression of P-gp was increased by CPT-11 treatment. Therefore, the contribution of P-gp, which is conceivably affected by transcellular absorption, may have decreased. This explanation is consistent with the clinical observation that the transcellular absorption was decreased whereas paracellular absorption was increased by chemotherapy treatment [16].

The inhibition extent of CAM in the intestine is determined by the intestinal concentration. In this study, the concentration of CAM in the gastrointestinal tract was considered to exceed 750 μg/mL based on the dosage of CAM (10 mg/kg) and the fluid volume of the rat gastrointestinal tract under fasted conditions (3.2 mL) [17]. Since this exceeds the in vitro IC_50_ of CAM to the DGX transport by P-gp (0.088 μg/L), [18] the dose of CAM used in this study was considered to be sufficient to inhibit intestinal P-gp. Indeed, CAM treatment increased the C_max_ and BA values of DGX in the control rats by 175 and 45%, respectively.

Solubility and permeability are other important factors affecting the intestinal absorption of drugs. In the CPT-11-treated group, the fluid volume in the small intestine might have increased due to the presence of severe diarrhea and reduced the DGX concentration in the intestinal tract, which could have impaired the absorption of the drug. Tanaka et al. reported that an increased luminal fluid volume might impair the absorption of atenolol, a Biopharmaceutics Classification System Class III drug (high solubility and low permeability), but not that of metoprolol (Class I, high solubility and high permeability) [19]. Since DGX is classified as a highly permeable drug, [20] the altered fluid volume might not have affected its permeability. Additionally, we administered DGX as elixir solution to minimize the effect of solubility. However, in the CPT-11-treated rats, the absorptive surface area was decreased. As it was found to be the case for acetaminophen in our previous study, [10] the decreased surface area might have limited the absorption of DGX, and changes in fluid volume might have a relatively important impact on drug absorption of DGX in rats with gastrointestinal damage. Other possible factors that might explain the discrepancy between the expression and activity of P-gp include the depletion of ATP and changes in the intracellular location of P-gp. Regarding the inhibition potency of CAM, the concentration of CAM in the gastrointestinal tract was considered to exceed 750 μg/mL, which is more than 1000-fold higher than the IC_50_, previously reported [18]. Even if the intestinal fluid volume was increased 3-fold by CPT-11-treatment, the intestinal concentration of CAM is considered to be still high enough to inhibit P-gp thoroughly. To support the results of CAM study and to further investigate P-gp-independent effects of intestinal environment on DGX absorption, further study using *Mdr1* knockout rats might be useful.

DGX is not only a substrate of P-gp, but also a substrate of an intestinal uptake transporter, Oatp1a5 [21]. In this study, the expression of Oatp1a5 might have been altered by CPT-11 treatment to affect the absorption of DGX. However, CAM, also known as a potent inhibitor of Oatp1a5 with the K_i_ value of 2.4 μM [22], failed to decrease the absorption of DGX in any conditions, suggesting that the contribution of Oatp1a5 is negligible. Therefore, the absorption of DGX in the presence of CAM is considered to reflect the passive absorption without the function of transporters.

We also made an attempt to carry out everted sac and closed loop intestinal perfusion study in CPT-11-treated rats. However, the intestinal tract was too fragile so that the results showed the intestine became quite leaky or torn. Further study using vesicles prepared from the CPT-11-treated intestine may be required to explain the discrepancy between the protein level and function of P-gp.

Although the primary goal of this study was to quantitatively evaluate the change in the gastrointestinal absorption of DGX, the systemic clearance of the drug was also reduced by CPT-11 treatment. Previously*,* we have reported that the elimination of DABE was delayed in rats treated with CPT-11, and concluded that impaired bile excretion and/or changes in the distribution volume of DABE induced by CPT-11 treatment might have been responsible because the levels of markers of both renal and liver disorders remained unchanged in the CPT-11-treated rats [10]. Although the bile excretion function via P-gp transport has not been examined in this model, CAM administration decreased the CL_iv_ approximately 10% in both control and CPT-11 treated group. This corresponds to the percentage of bile excretion to the total clearance of DGX in rats, [23] thus the decreased clearance does not attribute to renal, liver or bile excretion disorders. Other factors, such as hepatic blood flow, and protein binding, might have been affected by CPT-11 treatment. Further studies are required to clarify the mechanism responsible for the decreased systemic clearance of DGX seen in the CPT-11-treated rats.

## Conclusion

CPT-11-induced gastrointestinal damage decreased the intestinal absorption of DGX. However, in contrast to the control group, intestinal P-gp inhibition by CAM did not increase the absorption of DGX in the CPT-11 treated group. These results suggest the possibility that P-gp is not responsible for the decreased absorption of DGX, although further studies are necessary to clarify the mechanism.

## Data Availability

All data generated or analyzed during this study are included in this published article.

## Abbreviations

AUC: Area under the plasma concentration curve
AUMC: Area under the first moment curve
BA: Bioavailability
Bcrp: Breast cancer resistance protein
CAM: Clarithromycin
C_max_: Maximum plasma concentration
CMC: Carboxymethyl cellulose
C_0_: Plasma concentration at time zero
CPT-11: Irinotecan
DABE: Dabigatranetexilate
DGX: Digoxin
D: Dose
MRT: Mean residence time
P-gp: P-glycoprotein
Pept1: Peptide transporter 1
PK: Pharmacokinetics
SN-38: 7-ethyl-10-hydroxycamptothecin
